# Incidence rates of retinal vascular occlusive diseases from 2011 to 2020 in South Korea: a nationwide cohort study

**DOI:** 10.1186/s12886-024-03397-7

**Published:** 2024-03-22

**Authors:** Shin Hyeong Park, Bum Jun Kim, Ji Hye Kim, Seung Chan Kim, Rock Bum Kim, Yong Seop Han

**Affiliations:** 1 Department of Ophthalmology, Gyeongsang National University Changwon Hospital, #11 Samjeongja-ro, Seongsan- gu, Changwon, 51472 Korea; 2 Department of Biostatistics, Clinical Trial Center, Biomedical Research Institute, Pusan National University Hospital, Busan, Korea; 3 Regional Cardiocerebrovascular Disease Center, Gyeongsang National University Hospital, Jinju, Korea; 4 Department of Preventive Medicine, Institute of Health Sciences, Gyeongsang National University College of Medicine, Jinju, Korea; 5 Department of Ophthalmology, College of Medicine, Gyeongsang National University, Jinju, Korea; 6 Gyeongsang Institute of Health Sciences, Gyeongsang National University, Jinju, Korea

**Keywords:** Epidemiology, Incidence rate, Retinal artery occlusion, Retinal vascular occlusion, Retinal vein occlusion

## Abstract

**Background:**

Retinal vascular occlusions, including retinal vein occlusion and retinal artery occlusion, are common causes of visual impairment. In order to evaluate the national medical burden and help improve ophthalmic health care policy planning, we investigated the incidence of retinal vascular occlusive diseases from 2011 to 2020 in Korea.

**Methods:**

This study is a nationwide population-based retrospective study using data from the Korea national health claim database of the Health Insurance Review and Assessment (HIRA) service. We identified retinal vascular occlusive diseases registered from January 1, 2009, to December 31, 2020, according to the retinal vascular occlusion code (H34) and its sub-codes from international classification of disease, tenth revision diagnosis code. We used data from the entire Korean population based on the 2015 census of the population in Korea to calculate standardized incidence rates.

**Results:**

We identified 348,775 individuals (male, 161,673 [46.4%]; female, 187,102 [53.6%]) with incident retinal vascular occlusion (H34), 10,451 individuals (males, 6,329 [60.6%]; females, 4,122 [39.4%]) with incident central retinal artery occlusion (H34.1), and 252,810 individuals (males, 114,717 [45.4%]; females, 138,093 [54.6%]) with incident retinal vein occlusion (H34.8) during the 10-year study period. The weighted mean incidence rate of retinal vascular occlusion was 70.41 (95% CI, 70.18–70.65) cases/100,000 person-years. The weighted mean incidence rate of central retinal artery occlusion was 2.10 (95% CI, 2.06–2.14) cases/100,000 person-years. The weighted mean incidence rate of retinal vein occlusion was 50.99 (95% CI, 50.79–51.19) cases/100,000 person-years.

**Conclusion:**

The total retinal vascular occlusion and retinal vein occlusion showed a decreasing trend until 2020. However, the central retinal artery occlusion decreased until 2014 and remained stable without a significant further decline until 2020. The incidence of total retinal vascular occlusion and retinal vein occlusion was higher in females than in males, while the incidence of central retinal artery occlusion was higher in males. All retinal vascular occlusive diseases showed an increasing incidence with older age; the peak age incidence was 75–79 years for total retinal vascular occlusion and retinal vein occlusion, and 80–85 years for central retinal artery occlusion.

## Introduction

Retinal vascular occlusions, including retinal vein occlusion (RVO) and retinal artery occlusion (RAO), are common causes of visual impairment. The two forms of occlusion have different pathophysiology, systemic implications, and management; however, both are associated with increased age and cardiovascular risk factors such as hypertension, diabetes, and dyslipidemia [1–4]. Their symptoms can vary greatly, from minor visual discomfort to complete blindness, depending on the location and extent of the occlusion. RAO, including branch RAO (BRAO) and central RAO (CRAO), require specific systemic assessments for diagnosis and treatment. Fundamentally, RAO must be distinguished from venous occlusion, but mixed forms may exist, and an unspecified retinal vascular occlusion International Classification of Disease, Tenth Revision (ICD-10) diagnosis code has been used clinically [5]. Previously, some studies have investigated the incidence of CRAO and RVO in the Republic of Korea until 2015 [6, 7].; however, they did not investigate the overall incidence of retinal vascular occlusion or unspecified retinal vascular occlusion cases. In this study, we investigated the incidence of CRAO and RVO extending the research period until 2020. We also evaluated the incidence of overall retinal vascular occlusion using ICD-10 H34 code, which integrates all types of retinal vascular occlusion. The H34 code consists of the retinal vascular occlusion code (H34) and its subcodes, transient RAO (TRAO) (H34.0), CRAO (H34.1), other RAOs (H34.2), other retinal vascular occlusions (H34.8), and retinal vascular occlusion, unspecified (H34.9), from ICD-10. The findings derived from this study may help assess the public health burden associated with retinal vascular occlusion and improve ophthalmic health care policy planning.

## Methods

### Ethical statement

The study protocol was reviewed and approved by the Institutional Review Board of Gyeongsang National University, Changwon Hospital (approval no. GNUCH 2021-08-004) and was conducted in accordance with the tenets of the Declaration of Helsinki. The requirement for written informed consent was waived by the Institutional Review Board of Gyeongsang National University, Changwon Hospital (approval no. GNUCH 2021-08-004) because this study analyzed secondary data from the Korea national health claim database of the Health Insurance Review and Assessment (HIRA) service. The dataset did not include personal information such as patient name, social security number, address, or phone number. Only non-identifiable publicly available aggregate results were released for public research.

### HIRA and database

We used the South Korea health claims data recorded from January 1, 2009, to December 31, 2020, by the HIRA service. As of 2022, the total population of Korea was 51.63 million; of this, approximately 97% are enrolled in the Korean National Health Insurance (NHI) scheme, which is a compulsory health insurance. The claims, reviewed by the HIRA, include data on diagnoses, procedures, prescription records, demographic information, and direct medical costs. The HIRA also reviews claims from the Medical Assistance Program and Medical Care for Patriots and Veterans Affairs Scheme, which cover the medical expenses of NHI uninsured Korean population. Therefore, the HIRA database covers the entire Korean population and contains all Korean medical claims [8]. Patients in the HIRA are identified by their Korean Resident Registration Number, which is a unique identification number assigned to each Korean resident at birth. This ensures that there are no duplications or omissions when accessing the data [9].

### Identification of patients with incident retinal vascular occlusions

We identified retinal vascular occlusive diseases registered from January 1, 2009, to December 31, 2020, according to the Retinal vascular occlusion code (H34) and its sub-codes; TRAO (H34.0), CRAO (H34.1), other RAOs (H34.2), other retinal vascular occlusions (H34.8), and unspecified retinal vascular occlusion (H34.9) from ICD-10. We were not permitted to access the HIRA database for 2008 or earlier; therefore, we could not exclude retinal vascular occlusion cases diagnosed before 2009. Hence, we excluded cases with retinal vascular occlusion diagnostic codes between 2009 and 2010 to remove any potential preexisting cases of retinal vascular occlusion. All remaining patients had a disease-free period of at least 2 and 11 years before the index data; accordingly, they were considered to have had incident cases of retinal vascular occlusion [7].

### Statistical analysis

Statistical analyses were performed using R software version 4.2.1 (R Core Team. R Foundation for Statistical Computing, Vienna, Austria, 2022) and SAS software version 9.4 (SAS Institute, Inc.; Cary, NC, 2015). We calculated the yearly unadjusted retinal vascular occlusive disease incidence rates from 2011 to 2020 using the number of retinal vascular occlusive disease cases identified and the corresponding midyear population. The Korean population for each year was obtained from resident registration data in Korea (available at http://kosis.kr; accessed May 30, 2022), categorized into 5-yearly age groups and by sex. Next, we applied the direct method of standardization to estimate the incidence rates, standardized for age for each study year, using the 2015 Census of Population in Korea (available at http://kosis.kr, accessed May 30, 2022) as the standard population. Using these estimated standardized incidence rates, we calculated the weighted mean annual incidence rates of retinal vascular occlusive diseases from 2011 to 2020 and estimated the 95% confidence intervals (CIs) for the incidence rates per 100,000 person-years based on the Poisson distribution. Statistical analysis referred to the method used in previous studies [6, 7].

## Results

### Retinal vascular occlusion (H34)

We identified 348,775 individuals (male, 161,673 [46.4%]; female, 187,102 [53.6%]) with incident retinal vascular occlusion(H34) during the 10-year (2011-2020) study period (Table 1).

**Table 1:** Frequencies and incidence rates of retinal vascular occlusion in the Korean population from 2011 to 2020

Age group (years)	Korean population^a^			Total incidents		Incidents among male		Incidents among female		Male to female ratio
	Total residents	Male	Female	No.	Incidence rate (95% CI)^b^	No.	Incidence rate (95% CI)^b^	No.	Incidence rate (95% CI)^b^	
0–4	2235397	1147126	1088271	144	0.64 (0.54 to 0.76)	75	0.65 (0.51 to 0.82)	69	0.63 (0.49 to 0.8)	1.032
5–9	2252950	1162087	1090863	198	0.88 (0.76 to 1.01)	93	0.8 (0.65 to 0.98)	105	0.96 (0.79 to 1.17)	0.831
10–14	2418360	1257902	1160458	390	1.61 (1.46 to 1.78)	224	1.78 (1.55 to 2.03)	166	1.43 (1.22 to 1.67)	1.245
15–19	3170545	1657722	1512823	1139	3.59 (3.39 to 3.81)	630	3.8 (3.51 to 4.11)	509	3.37 (3.08 to 3.67)	1.130
20–24	3385936	1808857	1577079	1692	5 (4.76 to 5.24)	875	4.84 (4.52 to 5.17)	817	5.18 (4.83 to 5.55)	0.934
25–29	3027896	1581887	1446009	2416	7.98 (7.67 to 8.31)	1278	8.08 (7.64 to 8.54)	1138	7.87 (7.42 to 8.34)	1.027
30–34	3611034	1854905	1756129	3977	11.02 (10.68 to 11.37)	2291	12.36 (11.86 to 12.88)	1686	9.61 (9.15 to 10.07)	1.287
35–39	3783589	1927388	1856201	7549	19.97 (19.52 to 20.43)	4491	23.33 (22.65 to 24.02)	3058	16.49 (15.91 to 17.08)	1.415
40–44	4215921	2142101	2073820	13751	32.67 (32.13 to 33.22)	8067	37.73 (36.91 to 38.56)	5684	27.45 (26.74 to 28.17)	1.375
45–49	4266941	2151070	2115871	22328	52.47 (51.78 to 53.16)	12421	57.91 (56.9 to 58.94)	9907	46.94 (46.02 to 47.87)	1.234
50–54	4145976	2094318	2051658	33660	81.53 (80.66 to 82.4)	17230	82.62 (81.39 to 83.86)	16430	80.42 (79.19 to 81.66)	1.027
55–59	3863095	1922796	1940299	44725	116.44 (115.36 to 117.52)	21973	114.91 (113.4 to 116.44)	22752	117.95 (116.42 to 119.49)	0.974
60–64	2758941	1348273	1410668	48232	176.3 (174.73 to 177.88)	22862	170.95 (168.74 to 173.18)	25370	181.42 (179.19 to 183.66)	0.942
65–69	2117875	1015463	1102412	48461	231.47 (229.41 to 233.54)	22371	222.74 (219.83 to 225.68)	26090	239.51 (236.62 to 242.44)	0.930
70–74	1760932	789607	971325	47641	274.35 (271.89 to 276.82)	20185	258.9 (255.34 to 262.49)	27456	286.94 (283.55 to 290.35)	0.902
75–79	1356014	550684	805330	39617	296.32 (293.41 to 299.25)	15116	278.06 (273.65 to 282.53)	24501	308.83 (304.98 to 312.72)	0.900
80–84	810891	275462	535429	22553	281.69 (278.02 to 285.39)	8082	297.23 (290.78 to 303.78)	14471	273.7 (269.26 to 278.2)	1.086
85–89	371527	98367	273160	8300	225.58 (220.76 to 230.49)	2771	285.08 (274.56 to 295.89)	5529	204.23 (198.88 to 209.68)	1.396
90–94	124111	28565	95546	1737	140.81 (134.26 to 147.59)	546	192.76 (176.93 to 209.63)	1191	125.32 (118.31 to 132.65)	1.538
≥ 95	27732	5259	22473	265	95.94 (84.74 to 108.22)	92	176.11 (141.97 to 215.99)	173	77.25 (66.16 to 89.65)	2.280
Total	49705663	24819839	24885824	348775	70.41 (70.18 to 70.65)	161673	65.35 (65.03 to 65.67)	187102	75.47 (75.13 to 75.81)	0.866

*No.* number, *CI *confidence interval

^a^Korean population was based on the 2015 census data from the Korean Statistical Information Service

^b^Incidence rate was measured as cases per 100,000 person-years

The weighted mean incidence rate of retinal vascular occlusion during the 10 years was 70.41 (95% CI, 70.18 - 70.65) cases/100,000 person-years). By sex, these were 65.35 (95% CI, 65.03 to 65.67) and 75.47 (95% CI, 75.13 to 75.81) cases/100,000 person-years, in males and females, respectively.

**Fig. 1:**
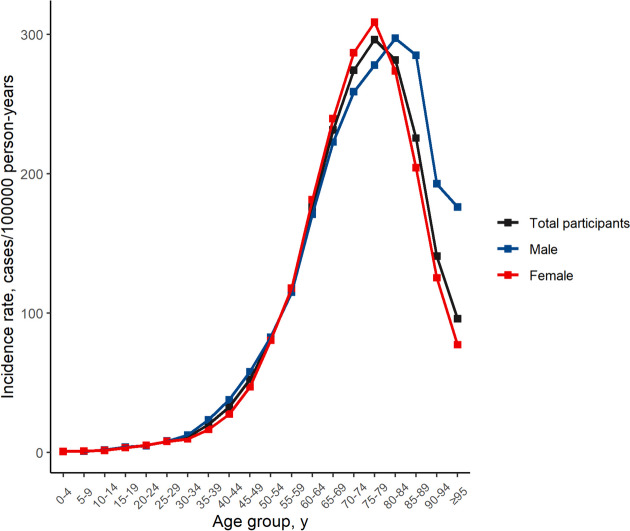
Total incidence rates by age group in retinal vascular occlusion in the Korean population from 2011 to 2020

The incidence rate increased considerably with increasing age, until age 75–79 years, with an incidence rate of 296.32 (95% CI, 293.41–299.25) cases/100,000 person-years. By sex, the highest incidence rate occurred in females aged 75–79 years (308.83; 95% CI, 304.98-312.72) and males aged 80–84 (297.23; 95% CI, 290.78-303-78) cases/100,000 person-years (Table 1, Fig. 1).

Overall, the male-to-female incidence ratio was 0.87. In most age groups, the incidence of retinal vascular occlusion was higher in females than males (Table 1). Males had a higher incidence of retinal vascular occlusion incidence than females among individuals aged 0–4, 10–19, 25–54, and > 79 years (Table 1).

**Fig. 2:**
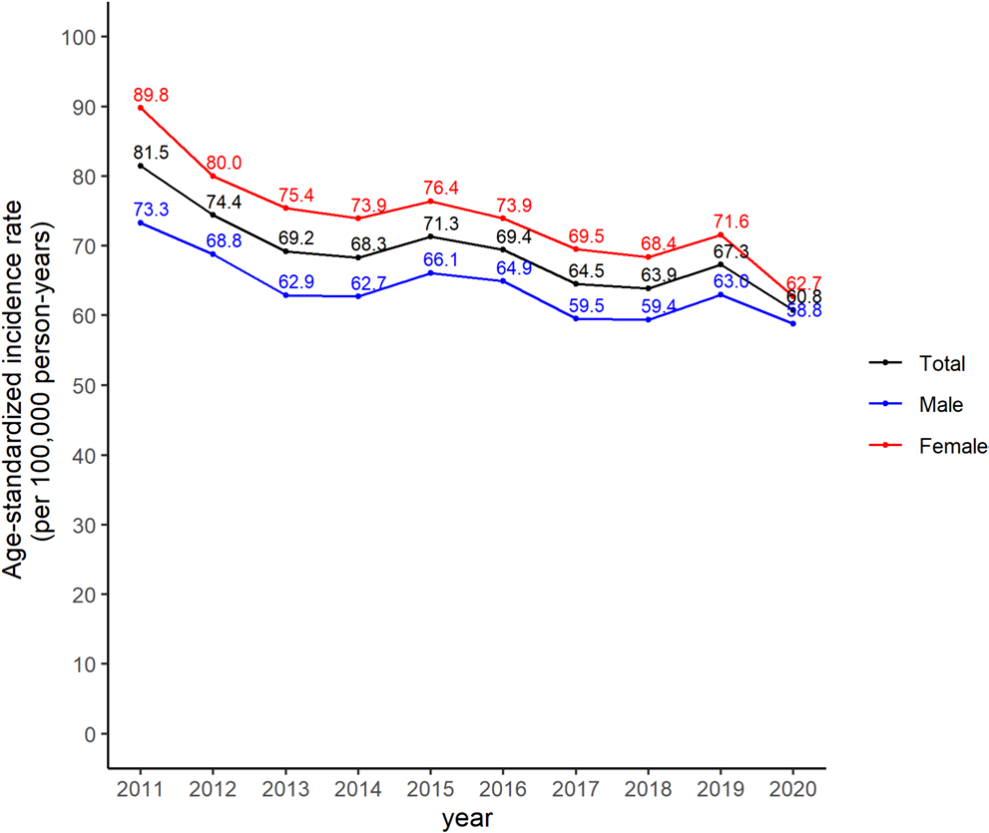
The age-standardized incidence rates of retinal vascular occlusion in the Korean population from 2011 to 2020

The overall trend of the age-standardized incidence rates of retinal vascular occlusion decreased from 2011 to 2020 in Korea. There were slight increases in 2015 and 2019 compared with the previous years, but the overall trend was not significantly different. Females had a higher incidence than males in all periods (Fig. 2).

### CRAO (H34.1)

We identified 10,451 individuals (males, 6,329 [60.6%]; females, 4,122 [39.4%]) with incident CRAO (H34.1) during the 10-year (2011-2020) study period (Table 2).

**Table 2:** Frequencies and incidence rates of central retinal artery occlusion in the Korean population from 2011 to 2020

Age group (years)	Korean population^a^			Total incidents		Incidents among male		Incidents among female		Male to female ratio
	Total residents	Male	Female	No.	Incidence rate (95% CI)^b^	No.	Incidence rate (95% CI)^b^	No.	Incidence rate (95% CI)^b^	
0–4	2235397	1147126	1088271	4	0.02 (0 to 0.05)	2	0.02 (0 to 0.06)	2	0.02 (0 to 0.07)	0.944
5–9	2252950	1162087	1090863	4	0.02 (0 to 0.04)	4	0.03 (0.01 to 0.09)	0	0 (0 to 0.03)	NA
10–14	2418360	1257902	1160458	12	0.05 (0.03 to 0.09)	7	0.06 (0.02 to 0.12)	5	0.04 (0.01 to 0.1)	1.302
15–19	3170545	1657722	1512823	39	0.12 (0.09 to 0.17)	24	0.14 (0.09 to 0.22)	15	0.1 (0.06 to 0.16)	1.465
20–24	3385936	1808857	1577079	56	0.16 (0.12 to 0.22)	23	0.13 (0.08 to 0.19)	33	0.21 (0.14 to 0.29)	0.608
25–29	3027896	1581887	1446009	99	0.33 (0.27 to 0.4)	48	0.3 (0.22 to 0.4)	51	0.35 (0.26 to 0.46)	0.858
30–34	3611034	1854905	1756129	133	0.37 (0.31 to 0.44)	76	0.41 (0.32 to 0.51)	57	0.32 (0.25 to 0.42)	1.262
35–39	3783589	1927388	1856201	181	0.48 (0.41 to 0.55)	111	0.58 (0.47 to 0.69)	70	0.38 (0.29 to 0.48)	1.528
40–44	4215921	2142101	2073820	313	0.74 (0.66 to 0.83)	182	0.85 (0.73 to 0.98)	131	0.63 (0.53 to 0.75)	1.345
45–49	4266941	2151070	2115871	445	1.04 (0.95 to 1.15)	272	1.26 (1.12 to 1.42)	173	0.82 (0.7 to 0.95)	1.546
50–54	4145976	2094318	2051658	704	1.7 (1.57 to 1.83)	438	2.09 (1.9 to 2.3)	266	1.3 (1.15 to 1.46)	1.613
55–59	3863095	1922796	1940299	1010	2.62 (2.46 to 2.78)	696	3.62 (3.36 to 3.9)	314	1.62 (1.44 to 1.81)	2.237
60–64	2758941	1348273	1410668	1178	4.27 (4.03 to 4.52)	815	6.05 (5.64 to 6.48)	363	2.57 (2.32 to 2.85)	2.349
65–69	2117875	1015463	1102412	1459	6.89 (6.54 to 7.25)	965	9.51 (8.92 to 10.13)	494	4.48 (4.1 to 4.89)	2.121
70–74	1760932	789607	971325	1666	9.46 (9.02 to 9.93)	1022	12.95 (12.17 to 13.77)	644	6.63 (6.13 to 7.16)	1.953
75–79	1356014	550684	805330	1614	11.91 (11.34 to 12.51)	896	16.28 (15.23 to 17.39)	718	8.92 (8.28 to 9.6)	1.825
80–84	810891	275462	535429	1003	12.38 (11.62 to 13.17)	517	18.78 (17.2 to 20.47)	486	9.08 (8.29 to 9.93)	2.068
85–89	371527	98367	273160	421	11.34 (10.28 to 12.47)	183	18.62 (16.02 to 21.52)	238	8.72 (7.64 to 9.9)	2.135
90–94	124111	28565	95546	100	8.06 (6.56 to 9.8)	43	15.06 (10.9 to 20.29)	57	5.97 (4.52 to 7.73)	2.524
≥ 95	27732	5259	22473	10	3.61 (1.73 to 6.63)	5	9.51 (3.09 to 22.19)	5	2.22 (0.72 to 5.19)	4.274
Total	49705663	24819839	24885824	10451	2.1 (2.06 to 2.14)	6329	2.55 (2.49 to 2.61)	4122	1.66 (1.61 to 1.71)	1.540

*No. *number, *CI *confidence interval, *NA *not applicable

^a^Korean population was based on the 2015 census data from the Korean Statistical Information Service

^b^Incidence rate was measured as cases per 100,000 person-years

The weighted mean incidence rate of CRAO during the 10 years was 2.10 (95% CI, 2.06 - 2.14) cases/100,000 person- years. By sex, these were 2.55 (95% CI, 2.49 to 2.61) and 1.66 (95% CI, 1.61 to 1.71) cases/100,000 person-years, in males and females, respectively.

**Fig. 3:**
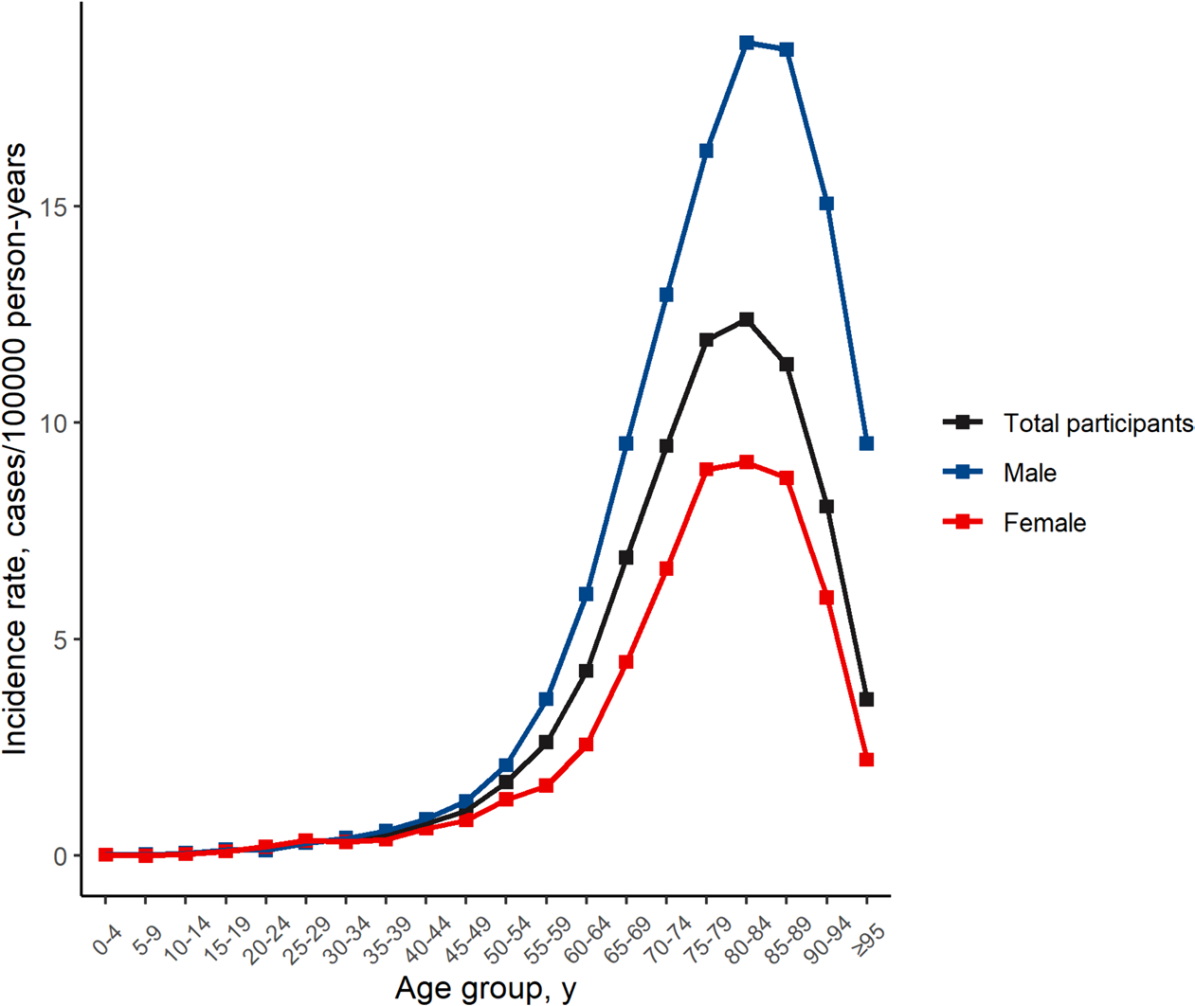
Total incidence rates by age group in central retinal artery occlusion in the Korean population from 2011 to 2020

The incidence rate increased considerably with age, until 80–84 years, with an incidence rate of 12.38 (95% CI, 11.62 to 13.17) cases/100,000 person-years. By sex, the highest incidence rate occurred in females (9.08; 95% CI, 8.29-9.93) and males (18.78; 95% CI, 17.20-20.47) cases/100,000 person-years, aged 80–84 years (Table 2, Fig. 3).

Overall, the male-to-female incidence ratio was 1.54. CRAO occurred more commonly in males than females across most age groups (Table 2). The incidence of CRAO was higher in males than in females among individuals aged 5–19 and > 29 years (Table 2).

**Fig. 4:**
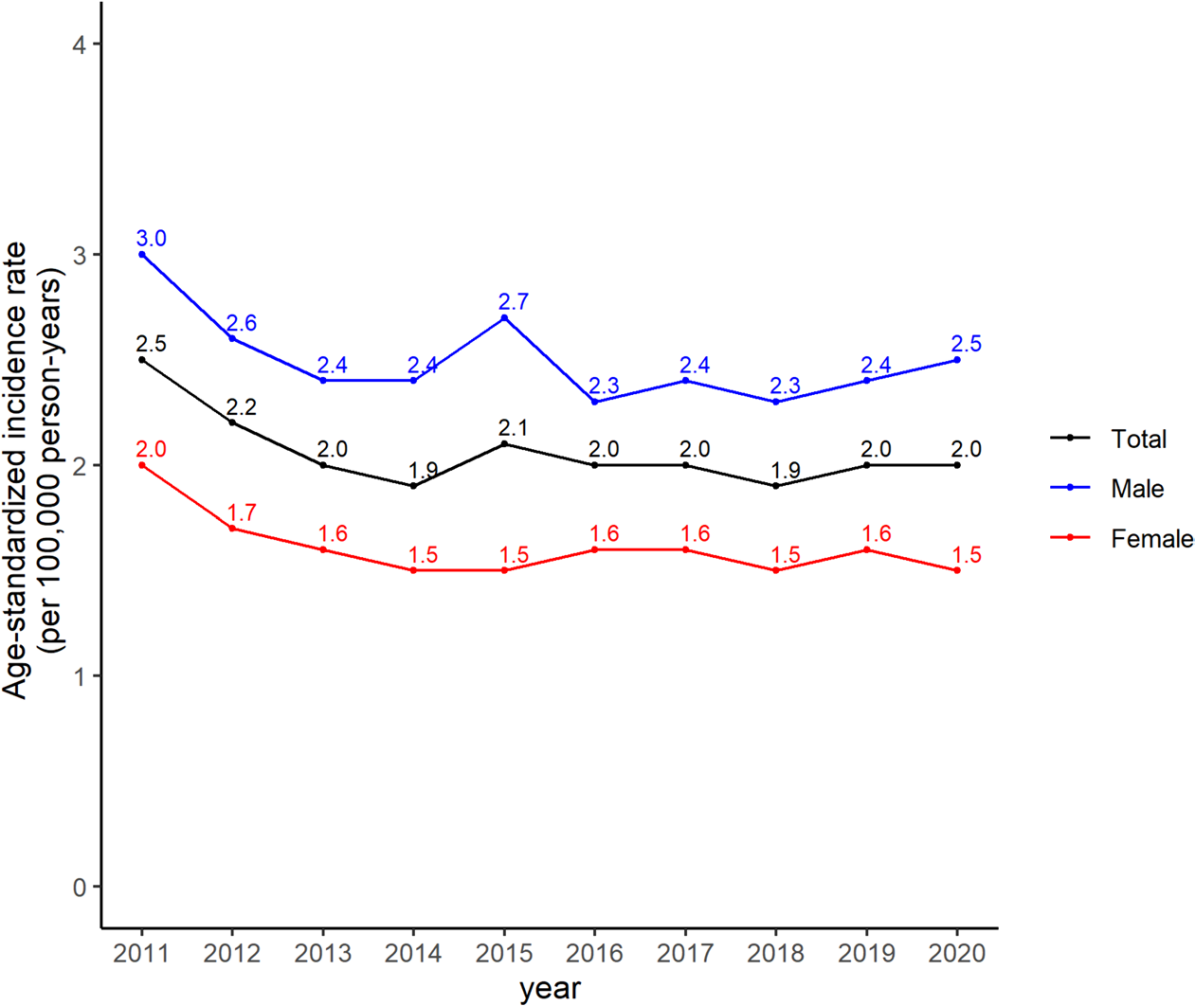
The age-standardized incidence rates of central retinal artery occlusion in the Korean population from 2011 to 2020

Until 2014, the age-standardized incidence of CRAO decreased. However, there was a slight increase in 2015 followed by a decrease in 2016. Thereafter, the age-standardized incidence rate remained stable without a further significant decline until 2020 ; the males had a higher incidence than females in all periods (Fig. 4).

### RVO (H34.8)

We identified 252,810 individuals (males, 114,717 [45.4%]; females, 138,093 [54.6%]) with incident RVO (H34.8) during the 10-year (2011-2020) study period (Table 3).

**Table 3:** Frequencies and incidence rates of retinal vein occlusion in the Korean population from 2011 to 2020

Age group (years)	Korean population^a^			Total incidents		Incidents among male		Incidents among female		Male to female ratio
	Total residents	Male	Female	No.	Incidence rate (95% CI)^b^	No.	Incidence rate (95% CI)^b^	No.	Incidence rate (95% CI)^b^	
0–4	2235397	1147126	1088271	56	0.25 (0.19 to 0.32)	27	0.23 (0.16 to 0.34)	29	0.27 (0.18 to 0.38)	0.883
5–9	2252950	1162087	1090863	91	0.4 (0.32 to 0.5)	38	0.33 (0.23 to 0.45)	53	0.49 (0.36 to 0.64)	0.673
10–14	2418360	1257902	1160458	196	0.81 (0.7 to 0.93)	108	0.86 (0.7 to 1.04)	88	0.76 (0.61 to 0.93)	1.133
15–19	3170545	1657722	1512823	580	1.83 (1.68 to 1.99)	315	1.9 (1.7 to 2.12)	265	1.75 (1.55 to 1.98)	1.084
20–24	3385936	1808857	1577079	919	2.71 (2.54 to 2.9)	478	2.64 (2.41 to 2.89)	441	2.8 (2.54 to 3.07)	0.945
25–29	3027896	1581887	1446009	1383	4.57 (4.33 to 4.82)	773	4.89 (4.55 to 5.24)	610	4.22 (3.89 to 4.57)	1.159
30–34	3611034	1854905	1756129	2428	6.73 (6.46 to 7)	1457	7.86 (7.46 to 8.27)	971	5.53 (5.19 to 5.89)	1.421
35–39	3783589	1927388	1856201	4920	13.01 (12.65 to 13.38)	3075	15.97 (15.41 to 16.54)	1845	9.94 (9.5 to 10.41)	1.605
40–44	4215921	2142101	2073820	9236	21.93 (21.49 to 22.38)	5655	26.43 (25.75 to 27.13)	3581	17.28 (16.72 to 17.86)	1.530
45–49	4266941	2151070	2115871	15737	36.95 (36.38 to 37.53)	8858	41.26 (40.41 to 42.13)	6879	32.56 (31.8 to 33.34)	1.267
50–54	4145976	2094318	2051658	24582	59.47 (58.73 to 60.22)	12609	60.39 (59.34 to 61.45)	11973	58.54 (57.49 to 59.6)	1.032
55–59	3863095	1922796	1940299	32919	85.57 (84.65 to 86.5)	16093	84.04 (82.74 to 85.34)	16826	87.1 (85.78 to 88.42)	0.965
60–64	2758941	1348273	1410668	35404	129.13 (127.79 to 130.48)	16424	122.53 (120.67 to 124.42)	18980	135.44 (133.52 to 137.38)	0.905
65–69	2117875	1015463	1102412	35544	169.26 (167.51 to 171.03)	15788	156.69 (154.26 to 159.16)	19756	180.86 (178.34 to 183.4)	0.866
70–74	1760932	789607	971325	35212	202.06 (199.95 to 204.18)	14098	180.15 (177.19 to 183.15)	21114	219.91 (216.95 to 222.9)	0.819
75–79	1356014	550684	805330	29464	219.6 (217.1 to 222.12)	10755	197.11 (193.41 to 200.88)	18709	235 (231.65 to 238.4)	0.839
80–84	810891	275462	535429	16672	207.55 (204.41 to 210.73)	5757	210.92 (205.51 to 216.44)	10915	205.82 (201.98 to 209.72)	1.025
85–89	371527	98367	273160	6022	163.24 (159.14 to 167.42)	1957	200.63 (191.84 to 209.72)	4065	149.8 (145.23 to 154.48)	1.339
90–94	124111	28565	95546	1257	101.73 (96.18 to 107.51)	383	134.87 (121.7 to 149.08)	874	91.84 (85.85 to 98.14)	1.468
≥ 95	27732	5259	22473	188	67.99 (58.62 to 78.43)	69	131.85 (102.59 to 166.87)	119	53.08 (43.97 to 63.52)	2.483
Total	49705663	24819839	24885824	252810	50.99 (50.79 to 51.19)	114717	46.32 (46.06 to 46.59)	138093	55.65 (55.35 to 55.94)	0.832

*No. *number, *CI *confidence interval

^a^Korean population was based on the 2015 census data from the Korean Statistical Information Service

^b^Incidence rate was measured as cases per 100,000 person-years

The weighted mean incidence rate of RVO during the 10 years was 50.99 (95% CI, 50.79 – 51.19) cases/100,000 person-years. By sex, the weighted mean incidence rates of RVO in males and females were 46.32 (95% CI, 46.06 to 46.59) and 55.65 (95% CI, 75.13 to 75.81) cases/100,000 person-years, respectively.

**Fig. 5:**
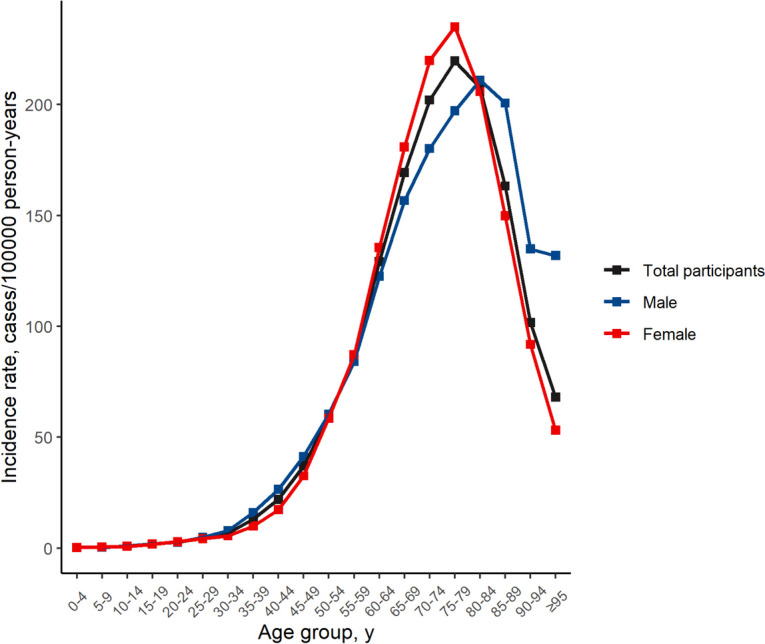
Total incidence rates by age group in retinal vein occlusion in the Korean population from 2011 to 2020

The incidence rate increased considerably with age, until age 75–79 years, with an incidence rate of 219.596 (95% CI, 217.10–222.12) cases/100,000 person-years. By sex, the highest incidence rate occurred among females aged 75–79 years (235.00; 95% CI, 231.65-238.40) and males aged 80–84 (210.92; 95% CI, 205.51-216-44) cases/100,000 person-years (Table 3, Fig. 5).

Overall, the male-to-female incidence ratio was 0.83. The females had a higher incidence rate than males in most age groups (Table 3). The males had a higher incidence of RVO than females among individuals aged 10–19, 25–54, and > 79 years (Table 3).

**Fig. 6:**
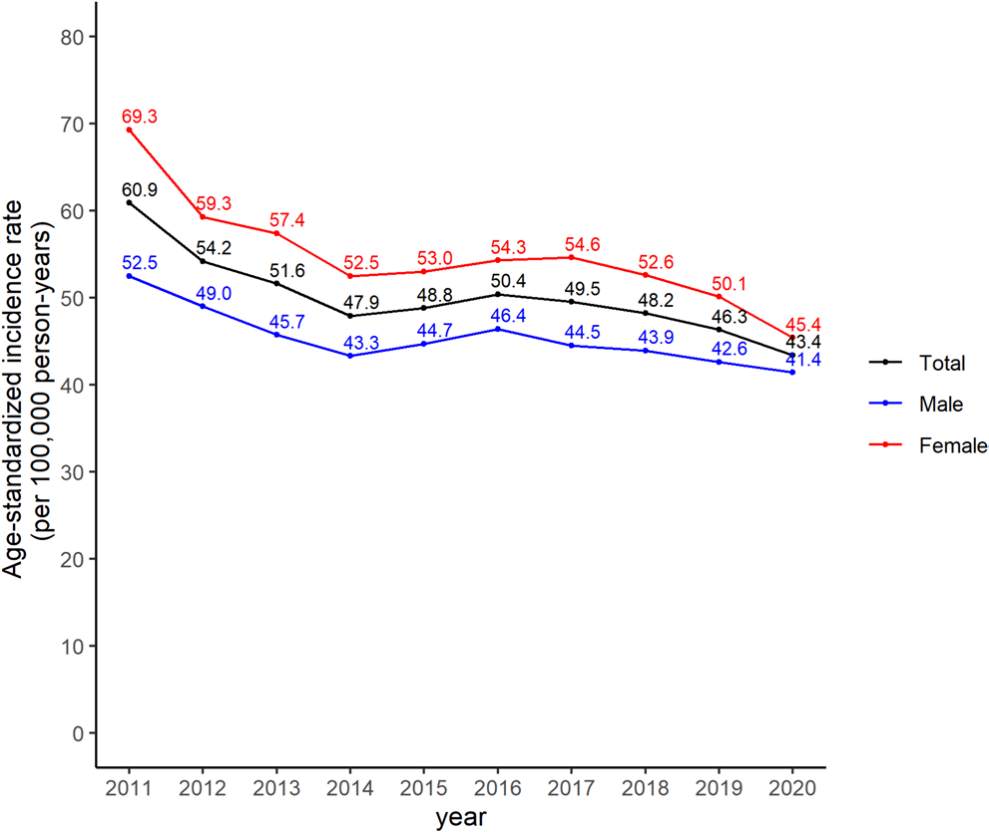
The age-standardized incidence rates of retinal vein occlusion in the Korean population from 2011 to 2020

The age-standardized incidence of RVO in Korea showed an overall decreasing trend from 2011 to 2020. Although there was a slight increase in 2015-2016, it did not significantly affect the overall trend of RVO incidence in Korea. The females had a higher incidence than males in all periods (Fig. 6).

## Discussion

In this nationwide population-based cohort study, the overall trend of age-standardized incidence rates of retinal vascular occlusion and RVO decreased from 2011 to 2020 in Korea; females had a higher incidence than males in all periods. However, the age-standardized incidence rates of CRAO decreased through 2014 and remained similar until 2020 without further decline; females had a higher incidence than males in all periods. The incidence of RVO was significantly higher than that of CRAO.

RVOs are much more common than RAOs and have a better prognosis [4, 5, 10, 11]. The pathophysiology and systemic implications of the two forms of occlusions differ greatly, but both occur more commonly in the older population (aged >50 years) and are associated with cardiovascular risk factors [1–5]. Over the last decade, awareness, treatment, and control of risk factors for Cardiovascular diseases (CVDs) risk factors, such as hypertension, diabetes, and smoking, have generally improved in Korea [12–14]. With the better control of CVD risk factors, the incidence rates of both stroke and coronary heart disease also decreased in Korea, from 2006 to 2010 [15]. According to previous nationwide population-based cohort studies, CRAO and RVO incidence rates decreased from 2004 to 2015 in Korea [6, 7]. They suggested that since CVDs risk factors are also associated with CRAO and RVO, the decreasing trend in the incidence of CRAO and RVO may be related to the successful control of CVDs risk factors. They also expected this decreasing trend to continue [6, 7].

Compared with earlier studies, our study period was extended to 2020. From 2011 to 2020, the age-standardized incidence rates of total retinal vascular occlusion and RVO decreased. Although there were slight increases in 2015 and 2019 for total retinal vascular occlusion and 2015-2016 for retinal vein occlusion, the overall trend declined from 2011 to 2020. However, the age-standardized incidence rate of CRAO decreased until 2014, after which it increased slightly in 2015 and then decreased again in 2016, remaining stable without a significant further decline until 2020. Contrary to expectations of previous studies, the age-standardized incidence rate of CRAO has remained stagnant rather than continuously decreasing. Therefore, we searched for the latest trends in previous studies on how CVDs and their risk factors have changed. According to a study investigating the epidemiology of CVDs and their risk factors in South Korea from 1983 to 2018 [16], although Korea's CVD mortality rate is decreasing the most, globally, the burden of CVDs is still increasing because of the rapid aging of the population and increasing number of patients with prevalent CVDs, which is expected to continue into the future. Among the risk factors for CVD, hypertension control has significantly improved; however, there is scope for further improvement. Smoking rates are declining significantly but are still high for males and increasing for females. The incidence of obesity, diabetes, and hypercholesterolemia is increasing, and action is required to reverse the trend. As the older-aged population is increasing, especially those with complex risk factors and chronic diseases, managing high-risk groups will become an important task for preventing CVDs in Korea. Therefore, further retinal vascular occlusive disease reduction may be difficult unless CVDs and their risk factors are managed more effectively.

The incidence of retinal vascular occlusion increased significantly with age, peaking between the ages of 75 and 79 years, similar to the prevalence rate in systemic CVDs [7, 13, 17]. The incidence rates of RVO increased with age and peaked by age 75–79. This result is similar to those of a previous study [7]. Among all retinal vascular occlusive diseases, RVO accounted for the most significant proportion, and the peak age for RVO and total retinal vascular occlusive disease are similar. The incidence of CRAO also increased with age, peaking between the ages of 80–85. Compared to a previous study, the peak age of CRAO incidence was delayed from 75–79 to years to 80–85 years [6]. The reason for the lower incidence of retinal vascular occlusion in the oldest group, may be due to difficulties in detecting symptoms and using medical care because of old age. In addition, since CRAO has more pronounced symptoms than RVO, it is possible that the peak incidence was observed at a slightly older age. As the population ages, a similar trend is expected in the future, and the peak age is expected to be delayed.

Regarding the age-standardized incidence rates from 2011 to 2020, the incidence of retinal vascular occlusion and RVO was higher in females, while the incidence of CRAO was higher in males. When analyzed by age group, females had a higher incidence of retinal vascular occlusion than males among those aged 5–9, 20–24, and 55–79 years. Females had a higher incidence of RVO than males among individuals aged 0–9, 20–24, and 55–79 years. Among individuals aged 5-19 and >29 years, males had a higher incidence of CRAO than females. These results show a sex distribution similar to that reported in previous domestic studies [6, 7]. A previous study [7] explained the higher incidence of RVO in females aged 20–29 years to be due to an increased risk of arterial and venous thrombosis because of oral contraceptive usage as well as preeclampsia/eclampsia in pregnant women. In those aged > 50 years, it was explained that RVO incidence in females would be higher than that in males, due to the increased risk of CVD caused by hormonal changes due to menopausal transition in females. In addition, while smoking and drinking decreased in males, they increased in females. The relatively high rate of hospital visits by females was also a reason for their increased RVO incidence. The reason for the generally high CRAO rate in males than that in females remains unclear. According to studies examining sex differences in the incidence of CVDs in South Korea [16], the incidence of most CVDs such as stroke and ischemic heart disease is higher in males. Unlike other types of CVD, heart failure occurs more frequently in females than in males and shows rapidly increasing mortality, incidence, and prevalence trends. Males and females also exhibit different patterns of CVD risk factor prevalence. In males, exposure to various risk factors increases rapidly in middle age but shows little change after 60 years of age. However, exposure to multiple risk factors continues to increase in females throughout their lifetime. Thus, females aged > 70 years have more risk factors than males of the same age.

In America, a recent study [18] using (Intelligent Research in Sight (IRIS) registry) data between 2013 and 2017, including 1,251,476 retinal vascular occlusion cases, reported sex at the onset of RAO and RVO. Cases were categorized based on diagnosis codes as RAO, with subtypes of TRAO, partial RAO (PRAO), BRAO, and CRAO; and as RVO, with subtypes venous engorgement (VE), branch RVO (BRVO), and central RVO (CRVO). Females had slightly higher incidences of TRAO, VE, and BRVO, whereas males had higher incidences of PRAO, BRAO, CRAO, and CRVO. Previous studies have suggested that venous thromboembolism is more common in females aged < 55 years because of pregnancy, postpartum conditions, and the use of oral contraceptives. However, among older populations, cases tend to be more common in males because of CVD risk factors [19, 20]. To date, several epidemiological studies have been conducted on retinal vascular occlusion, but studies investigating drug use and comorbidities are yet to be conducted. In addition, epidemiological studies on factors other than for CVDs that can affect retinal vascular occlusion, such as thrombosis and vasculitis, are lacking. If such studies are conducted in the future, the relationship between retinal vascular occlusion and sex can be explained in greater detail.

Further research on the correlation between recently emerging diseases and retinal vascular occlusion is required. As the coronavirus disease 2019 (COVID-19) spread rapidly worldwide, the World Health Organization (WHO) declared an international public health emergency in January 2020. In March, it was declared a global pandemic [21]. COVID-19 is caused by a new type of coronavirus, severe acute respiratory syndrome coronavirus 2 (SARS-CoV-2). It has been reported that, in addition to respiratory infections, SARS-CoV-2 may cause coagulation activation and systemic inflammatory response abnormalities, resulting in thrombotic microangiopathy and venous or arterial thromboembolic complications in COVID-19 patients [22, 23]. In ocular manifestations, RAO and RVO may be associated with thromboembolic complications. This study investigated the 10-year incidence rates, including that in 2020. However, the number of COVID-19 patients in South Korea in 2020 was relatively small, and an effect of COVID-19 on retinal vascular occlusion is limited in this study. Additional research is needed to analyze the incidence of RVO, CRAO, and retinal vascular occlusion from 2021 to 2022 as well as patients with COVID-19.

Our study has several limitations. First, we used a different institutional database than the previous studies with which we compared; however, they were all nationwide cohort studies [6, 7]. And they used data from the Korean National Health Insurance Service (NHIS). On the other hand, our study used HIRA data. Almost all Koreans (97%) are enrolled in the NHI program, and most receive medical treatment at least once a year. Data are collected by the HIRA and the results of the review are sent to the NHIS. The data handled by NHIS and HIRA cover almost the entire Korean population and can be analyzed for various research purposes [24]. Because both institutions provide data for the entire population enrolled in the NHI, it is impossible to compare their exact figures, but the changes in the overall trend can be confirmed. Second, we could not access hospital-based medical records for confirmation of retinal vascular occlusion occurrences or review the clinical data. Therefore, the accuracy of the data may be lower owing to the potential of misclassification of the diagnoses. Third, because this study used a claims database, it was difficult to standardize the clinical characteristics and interobserver variability in the diagnosis of retinal vascular occlusion. Fourth, we couldn’t have data about other systemic vascular diseases. Additional studies might be needed to analyze the correlation between retinal vascular occlusion and systemic vascular disease.

In conclusion, this nationwide cohort study determined the nationwide incidence of retinal vascular occlusion over a 10-year period for the first time. We also updated the recent nationwide incidence rates of CRAO and RVO. The decreasing trend in the RVO incidence continues to follow the trend in previous studies until 2020. The total retinal vascular occlusion showed a decreasing trend over time. However, the CRAO decreased until 2014 and remained stable without a significant further decline until 2020. The incidence of total retinal vascular occlusion and RVO was higher in females than in males, while the incidence of CRAO was higher in males. All retinal vascular occlusive diseases showed an increasing incidence with older age; the peak age incidence was 75–79 years for total retinal vascular occlusion and RVO, and 80–85 years for CRAO. Further studies are required to evaluate the long-term changes in total retinal vascular occlusion, CRAO, and BRVO incidence rates and their association with vascular comorbidities in Korea.

## Data Availability

The data was acquired from HIRA, and they have not given their permission for researchers to share their data. Data requests can be made to HIRA at https://opendata.hira.or.kr/home.do.

## Abbreviations

BRAO: Branch retinal artery occlusion
BRVO: Branch retinal vein occlusion
CI: Confidence intervals
COVID-19: Coronavirus disease 2019
CRAO: Central retinal artery occlusion
CRVO: Central retinal vein occlusion
CVDs: Cardiovascular diseases
HIRA: Health Insurance Review and Assessment
ICD-10: International Classification of Disease, Tenth Revision
IRIS: Intelligent Research in Sight
NHI: National Health Insurance
NHIS: National Health Insurance Service
PRAO: Partial retinal artery occlusion
RAO: Retinal artery occlusion
RVO: Retinal vein occlusion
SARS-CoV-2: Severe acute respiratory syndrome coronavirus 2
TRAO: Transient retinal artery occlusion
VE: Venous engorgement
WHO: World Health Organization
